# Effect of Sensory Deprivation of Nasal Respiratory on Behavior of C57BL/6J Mice

**DOI:** 10.3390/brainsci11121626

**Published:** 2021-12-09

**Authors:** Yongji Zhu, Yujing Ye, Chenyang Zhou, Siqi Sun, Jingjing Zhang, Zixuan Zhao, Tingting Sun, Jing Li, Jing Yang, Weiyun Li, Shanshan Li

**Affiliations:** 1 Department of Basic Medicine, School of Medicine, Zhejiang University City College, Hangzhou 310015, China; zyj2901142505@163.com (Y.Z.); yyj000426@163.com (Y.Y.); zhoucy9086@163.com (C.Z.); ssq44747@163.com (S.S.); hata6r@163.com (J.Z.); zzx001110@163.com (Z.Z.); yangjing@zucc.edu.cn (J.Y.); 2 College of Food Science and Pharmaceutical Engineering, Zaozhuang University, Zaozhuang 277160, China; suntingting1218@126.com; 3 Institute of Neuroscience and Anatomy, School of Medicine, Zhejiang University, Hangzhou 310058, China; lijing851@zju.edu.cn

**Keywords:** nasal breathing, sensory deprivation of nasal respiratory, behavioral test battery, C57BL/6J mice

## Abstract

Nasal breathing is a dynamic cortical organizer involved in various behaviors and states, such as locomotion, exploration, memory, emotion, introspection. However, the effect of sensory deprivation of nasal respiratory breath (NRD) on behavior remain poorly understood. Herein, general locomotor activity, emotion, learning and memory, social interaction, and mechanical pain were evaluated using a zinc sulfate nasal irrigation induced nasal respiratory sensory deprivation animal model (ZnSO_4_-induced mouse model). In the open field test, the elevated O-maze test, and forced swim test, NRD mice exhibited depressive and anxiety-like behaviors. In memory-associated tests, NRD mice showed cognitive impairments in the hippocampal-dependent memory (Y maze, object recognition task, and contextual fear conditioning (CFC)) and amygdala-dependent memory (the tone-cued fear conditioning test (TFC)). Surprisingly, NRD mice did not display deficits in the acquisition of conditional fear in both CFC and TFC tests. Still, they showed significant memory retrieval impairment in TFC and enhanced memory retrieval in CFC. At the same time, in the social novelty test using a three-chamber setting, NRD mice showed impaired social and social novelty behavior. Lastly, in the von Frey filaments test, we found that the pain sensitivity of NRD mice was reduced. In conclusion, this NRD mouse model showed a variety of behavioral phenotypic changes, which could offer an important insight into the behavioral impacts of patients with anosmia or those with an impaired olfactory bulb (OB) (e.g., in COVID-19, Alzheimer’s disease, Parkinson’s disease, etc.).

## 1. Introduction

Respiration is an essential metabolic activity as it provides oxygen, which involves nasal breathing and mouth breathing. The respiratory drive is produced by conditional bursting pacemaker neurons in the brain stem [1]. However, scientists discovered that nasal breathing is not constant and can be altered by various emotions [2], cognitive states [3] and internal states [4]. Thus, respiration regulation is a complex process closely related to various behaviors and conditions.

Mammalian olfactory sensory neurons have dual functions as odor detectors and mechanical sensors for nasal breathing [5]. During nasal breathing, the airflow moves along the olfactory epithelium at the roof of the nasal cavity, triggering olfactory sensory neurons to respond to mechanical stimuli airflow via a cAMP cascade and to induce olfactory bulb (OB) neuronal oscillations, which lock to breathing cycles [5]. There is now substantial evidence in both rodents and humans that demonstrates a surprising cycle-by-cycle influence of nasal respiration on network activity throughout the olfactory system as well as much of the cerebral cortex, including primary olfactory (piriform) cortex [3], hippocampus [6], prefrontal cortex (PFC) [7,8] and somatosensory barrel cortex [9]. Respiration-entrained oscillations are a global brain rhythm and might aid long-range communication in the brain [10]. For example, during active behaviors, such as locomotion, sniffing, and exploration, PFC exhibits exclusive coupling between respiration and the 70–120-Hz gamma sub-band; and the hippocampus exhibits coupling of the 40–90-Hz and 110–160-Hz gamma sub-bands to respiration [7]. With removal of olfactory bulb, naris occlusion, or destruction of nasal mucosa, the respiration-related oscillations in the OB are significantly weakened [2,11]. At the same time, the coherence and cross-correlation of OB with PFC activities [2], hippocampus activities [12], and barrel cortex activities [9] are also significant reduced. Although nasal breathing is closely related to the activity of multiple brain regions, studies on behavioral effects of nasal respiratory sensory deprivation (NRD) are scarce.

C57BL/6J wild-type mouse is an inbred mouse that is widely used as a background strain for mutant mice. In this experiment, it was used as the NRD model mouse, which were treated with zinc sulfate solution in the nasal cavity, leading to impair the perception of nasal breathing, such as breathing rate, inhalation time, and exhalation time [11,13]. Broad behavioral phenotyping was then performed within one week after zinc sulfate irrigation, with the use of open field [14] and elevated O maze [15] for locomotor activity and anxiety-behavior assessment, forced swim for depression behavior [16], Y maze [17], novel object recognition [18] and contextual fear conditioning [19] for hippocampal-dependent memory, tone-cued fear conditioning for amygdala-dependent memory [20], social discrimination for social behavior [21], and von Frey filaments test for allodynia [22]. These behavioral tests were performed in a uniform manner following standardized protocols. Our results showed significant behavioral differences in almost all the tests, demonstrating the impacts of nasal breathing sensory deprivation on various behavior in C57BL/6J mice. The detailed characterization of behavioral changes related to nasal breathing sensory deprivation reinforces the importance of nasal breathing and provides a model reference for researchers in subsequent related studies.

## 2. Materials and Methods

### 2.1. Animals

A total of 104 wild-type C57BL/6J male mice aged 8–10 weeks were used. All the mice were group-housed under standard housing conditions with free access to food and water. All animal experiments complied with the ARRIVE guidelines and were conducted following the National Research Council’s Guide for the Care and Use of Laboratory Animals and the guidelines for the Care and Use of Laboratory Animals of Zhejiang University City College.

### 2.2. Nasal Irrigation of Zinc Sulfate

To prevent zinc sulfate (ZnSO_4_) from being inhaled into the lungs, the mice were held by the experimenter’s hand without anesthesia, then 20 μL of 0.17 M ZnSO_4_ or normal saline was slowly introduced into each naris of mice using a probe point (blunt) needle, 26 Gauge. Due to the regeneration of the olfactory epithelium, behavioral experiments were conducted within one week after zinc sulfate irrigation [13].

### 2.3. Behavioral Test Battery

**Figure 1:**
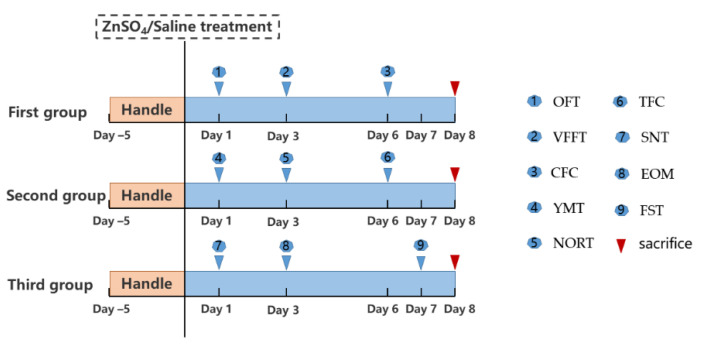
An illustration of the experimental timeline. Open field test, OFT; elevated O maze, EOM; forced swim test, FST; Y-maze test, YMT; novel object recognition test, NORT; contextual fear conditioning test, CFC; tone-cued fear conditioning test, TFC; social novelty test, SNT; von Frey filaments test, VFFT.

Mice were handled for five days before testing. If the mice could crawl freely on the experimenter’s hand and could be held in the experimenter’s hand without fear, they would be divided into three groups randomly, by using the standard = RAND () function in Microsoft Excel. Different behavioral experiments were assigned to three groups and named the first group, the second group, and the third group. Then, the three animal groups were randomly assigned to different experiment groups. The first group underwent in the following order: open field, von Frey filaments test, and the contextual fear conditioning; the second group was used successively in the Y maze, the novel object recognition test, and the cued fear conditioning test; and the third group was utilized in the social discrimination test, elevated O maze, and forced swim test (Figure 1). During the experiment, there were some precautions: (1) the interval between each behavioral experiment should be at least a day apart; (2) all behavioral tests were performed between 8:00 am and 8:00 pm; (3) one hour before testing, the mice were placed in the testing room for acclimatization to the room; (4) all behavior studies were conducted in different rooms with basically same environments; (5) after each experiment, the build-up of odor traces left by the previous mouse were removed with 75% ethanol to prevent interference with the next investigation; (6) all animals were euthanized with sodium pentobarbital approximately 24 h after the last behavioral assessment; and (7) if the data of individual mice was missing due to the dropout of equipment signal, these mice were excluded.

#### 2.3.1. Open Field Test (OFT)

**Figure 2:**
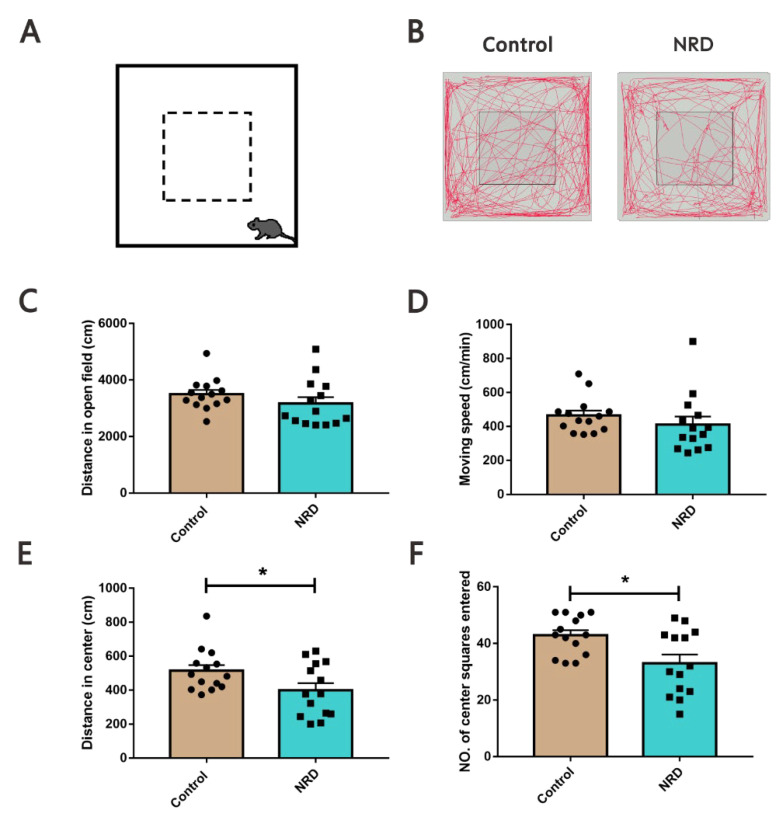
Normal locomotor activity and anxiety-like behavior in NRD mice. (**A**) Diagram of the open field apparatus (the peripheral (between solid and dotted lines) and central (inside the dotted line) regions). (**B**) Representation of the locomotion tracing for control and NRD mouse in the open field test. (**C**–**F**) Performances of the control and NRD mice in terms of distance in open field (**C**), moving speed in open field (**D**), in center zone (**E**), and number of center squares entered (**F**). * *p* < 0.05 as determined by unpaired Student’s *t*-test. Data are expressed as the means ± SEMs. *n* (control) = 14, *n* (NRD) = 14.

The OFT was performed to measure general locomotor activity and anxiety-behavior as previously described [14]. The custom-made open field apparatus consisted of a square arena (40 cm × 40 cm × 40 cm, length × width × height) that was virtually divided into a center field (center, 20 × 20 cm) and a periphery field (Figure 2A). Each mouse was allowed to explore the test area for 10 min. The test sessions were recorded by a video camera installed on the ceiling above the apparatus and analyzed using EthoVision tracking software (Noldus EthoVision XT10).

#### 2.3.2. Elevated O Maze (EOM)

**Figure 3:**
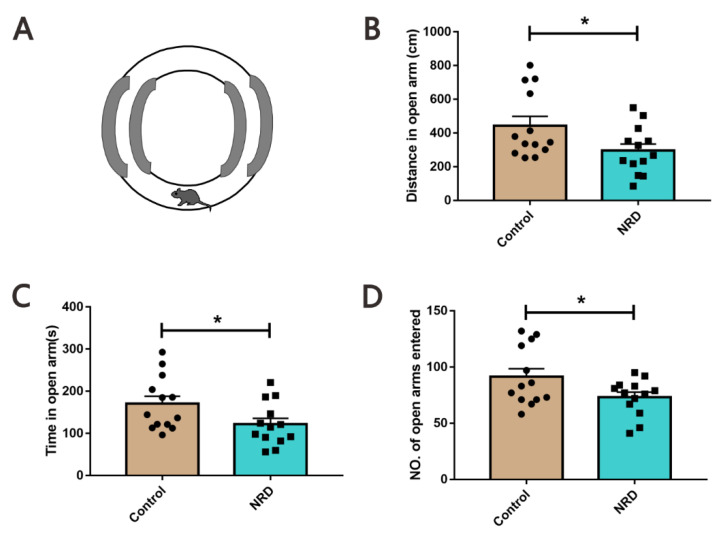
Anxiety-like behavior in NRD mice. (**A**) Diagram of the O-maze apparatus. (**B**–**D**) Performances of the control and NRD mice regarding the distance covered in open arms (**B**), time in open arms (**C**), and number of open arms entered (**D**). * *p* < 0.05 as determined by unpaired Student’s *t*-test. Data are expressed as the means ± SEMs. *n* (control) = 13, *n* (NRD) = 13.

The EOM was conducted to assess anxiety-behavior according to previous report [15]. The custom-made O maze (27 cm × 6 cm, inside diameter × width) consisted of two equal open arms and two closed arms (surrounded by a 12-cm high black wall) elevated 75 cm from the ground (Figure 3A). The mouse was placed at the boundary between the open and closed arms, was allowed to explore for 10 min, was tracked by a video camera installed on the ceiling above the O maze, and analyzed using EthoVision tracking software (Noldus EthoVision XT10).

#### 2.3.3. Forced Swim Test (FST)

**Figure 4:**
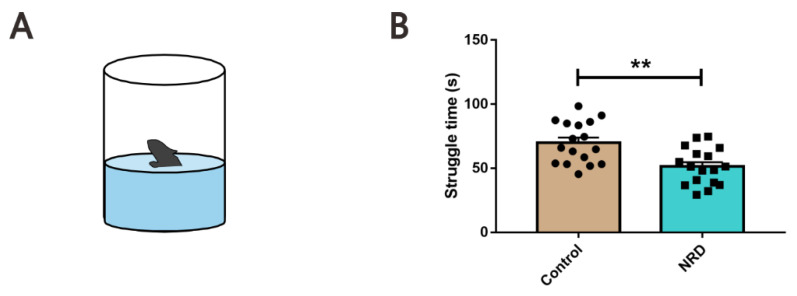
Decreased struggle time in NRD mice. (**A**) Diagram of the forced swim test. (**B**) Struggle times of the control and NRD mice in the forced swim test. ** *p* < 0.01 as determined by unpaired Student’s *t*-test. Data are expressed as the means ± SEMs. *n* (control) = 17, *n* (NRD) = 17.

The FST was conducted to measure depression-behavior as previously described [16]. Each mouse was individually placed in an open cylindrical container (14 cm × 34 cm, diameter × height) filled to a depth of 18 cm with 25 ± 1 °C water and allowed to swim for 6 min (Figure 4A). Immobility was defined as no movement other than movement required to balance the body and keep the head above water. The behavior of the mice was video-recorded by a camera located in front of the container, and the struggle time of 2–6 min was analyzed three times by an experienced investigator who blinded to the experiment and the average value was taken.

#### 2.3.4. Y-Maze Test (YMT)

**Figure 5:**
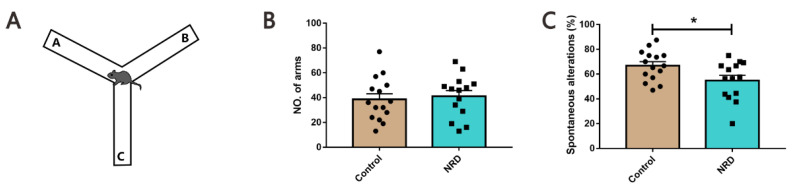
Impairment of Y-maze spontaneous alternation rate in NRD mice. (**A**) Diagram of the Y-maze working memory task. (**B**) The number of entries in all arm of the Y maze. (**C**) Spatial memory measured as alternation percentages in the Y maze. * *p* < 0.05 as determined by unpaired Student’s *t*-test. Data are expressed as the means ± SEMs. *n* (control) = 15, *n* (NRD) = 14.

The YMT was carried out to evaluate spatial working memory according to our previous report [23]. The custom-made Y-maze test was composed of three arms (35 cm × 6 cm × 15 cm, length × width × height) labeled A, B, and C (Figure 5A). The mice were placed at the end of arm A, facing the maze’s center, and were allowed to explore for 10 min. The sequence of arm entries and the total number of entries were recorded by a video camera installed on the ceiling above the Y maze for offline blind analysis. The three consecutive different choices of three arms were calculated as a correct alternation (i.e., ABC, BCA). The number of correct alternations was divided by the total number of triplets and multiplied by 100 to obtain Spontaneous Alternation (%).

#### 2.3.5. Novel Object Recognition Test (NORT)

**Figure 6:**
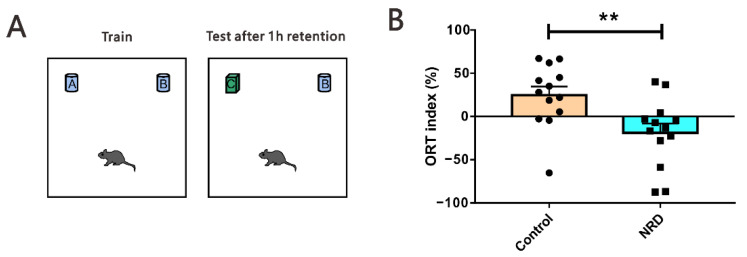
Defected cognition in object recognition memory in NRD mice. (**A**) Diagram of the novel object recognition test. (**B**) Performances of the control and NRD mice in the novel object recognition test. ** *p* < 0.01 as determined by unpaired Student’s *t*-test. Data are expressed as the means ± SEMs. *n* (control) = 13, *n* (NRD) = 13.

The NORT was performed to assess declarative memory according to a previous report [24]. The apparatus for the NORT was an open square chamber (40 cm × 40 cm × 40 cm, length × width × height) (Figure 6A). After a 10-min habituation period in the chamber, the mouse was allowed to explore two identical objects A and B (approximately 6 cm from the walls) for 10 min. After a retention period of 1 h, familiar object A was replaced with the novel object C, and the mouse was allowed to explore for 10 min. Sniffing time was defined as the time when the subject’s nose is within 2 cm of the object. The sniffing time spent on the new object and the old object was recorded in the last 10 min by EthoVision tracking software, and the NOR index was calculated as follows: (time to explore the novel object C time to explore old object B)/(the total time to explore two objects).

#### 2.3.6. Contextual Fear Conditioning Test (CFC)

**Figure 7:**
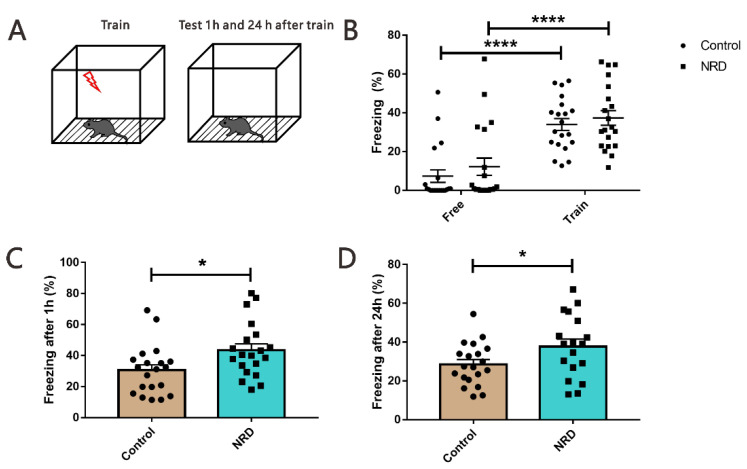
Enhanced contextual fear memory in NRD mice. (**A**) Diagram of the contextual fear conditioning test. (**B**) Performance of control mice and NRD mice before and during training. **** *p* < 0.0001 as determined by the two-way ANOVA. *n* (control) = 20, *n* (NRD) = 20. (**C**) Freezing behavior tests after 1 h of training. *n* (control) = 20, *n* (NRD) = 20. (**D**) Freezing behavior tests after 24 h of training. *n* (control) = 20, *n* (NRD) = 18. * *p* < 0.05 as determined by the unpaired Student’s *t*-test. Data are expressed as the means ± SEMs.

The CFC test was performed to evaluate contextual fear conditional learning and memory as previously described [20]. During the training phase, the mice were first allowed to move freely for 2 min and were given a 2-s, 0.3 mA foot shock 3 times with an interval of 20 s in between (Figure 7A-train). Finally, the mice stayed in the chamber for another 2 min after the shock. One and 24 h after the training, the mice were placed on the platform again for 5 min (without foot shock) (Figure 7A-test), and their freezing behaviors were analyzed.

#### 2.3.7. Tone-Cued Fear Conditioning Test (TFC)

**Figure 8:**
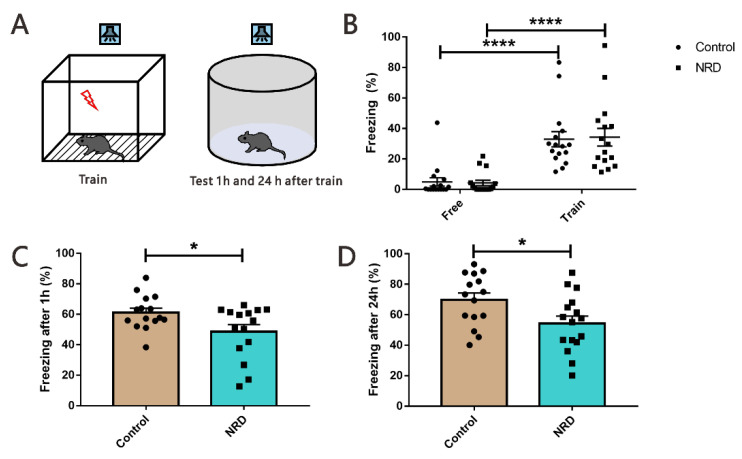
Reduced tone-cued fear memory in NRD mice. (**A**) Diagram of the cued fear conditioning test. (**B**) Performance of NRD mice and control mice before and during training. *n* (control) = 16, *n* (NRD) = 16. (**C**) Freezing behavior tests after 1 h of training. *n* (control) = 15, *n* (NRD) = 15. (**D**) Freezing behavior tests after 24 h of training. *n* (control) = 15, *n* (NRD) = 16. * *p* < 0.05, as determined by unpaired Student’s *t*-test; **** *p* < 0.0001 as determined by the two-way ANOVA. Data are expressed as the means ± SEMs.

The TFC was designed to assess tone-cued fear conditional learning and memory based on a previous report [20]. First, mice were trained within the same chamber with the contextual fear conditioning (Figure 8A-train). Training consisted of 2 min of free exploration, 3 tone-foot shock pairs (90 s apart, 30 s tone (80 dB, 4 kHz) co-terminating with a 2-s, 0.5 mA foot shock) and another 2-min layover. After 1 h and 24 h, to test the extent of fear, the trained mice were introduced in chamber 2 with a changed environment (shape, color, and floor), and the training process was repeated without giving foot shock (Figure 8A-test). The fear conditioning experiment was performed using the Fear Combined System (Panlab). The percentage of freezing time to tone alone was analyzed.

#### 2.3.8. Social Novelty Test (SNT)

**Figure 9:**
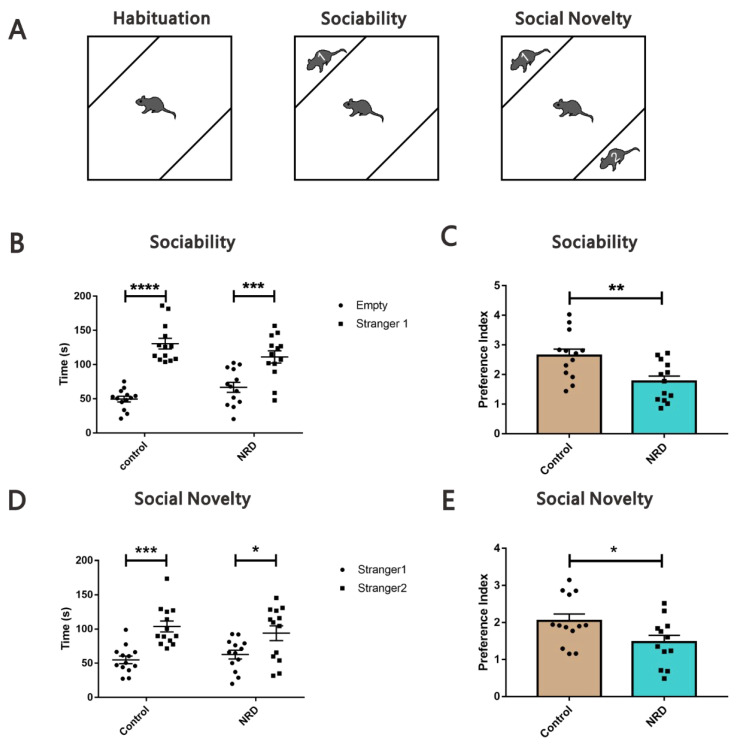
Both social behavior and social novelty behavior were impaired in NRD mice. (**A**) Diagram of the social novelty test. (**B**) Sniffing time of empty and stranger 1in the control and NRD mice during sociability session. * *p* < 0.05, *** *p* < 0.001, as determined by the two-way ANOVA. (**C**) Quantitative sociability assessment. ** *p* < 0.01 as determined by unpaired Student’s *t*-test. (**D**) Sniffing time of stranger 1 and stranger 2 in the control and NRD mice during social novelty session. *** *p* < 0.001, **** *p* < 0.0001, as determined by the two-way ANOVA. (**E**) Quantitative social novelty assessment. * *p* < 0.05 as determined by unpaired Student’s *t*-test. Data are expressed as the means ± SEMs. *n* (control) = 13, *n* (NRD) = 13.

The SNT test was performed to evaluate social behavior as previously described [21]. The three-chamber test (40 cm × 40 cm × 40 cm, length × width × height) consisted of a three-chamber apparatus including two mouse holders placed diagonally (Figure 9A). The trial comprised of three 10-min sessions without intertrial intervals (ITIs). In the first session (habituation), subject mice were allowed to acclimatize to the arena. In the second session (sociability), a never-before-met male mouse (stranger 1) was placed in one mouse holder while the other remained empty. In the third session (social novelty), the one stranger mouse remained unchanged, and another stranger mouse (stranger 2) was placed in the other mouse holder. The sniffing time was defined as the amount of time the subject mouse had its nose within 2 cm of a mouse holder. The test sessions were recorded by a video camera and analyzed using EthoVision tracking software (Noldus). A total of 52 C57BL/6J male strange mice (2–3 months of age) were used. Each mouse was used only once for each experiment.

#### 2.3.9. Von Frey Filaments Test (VFFT)

**Figure 10:**
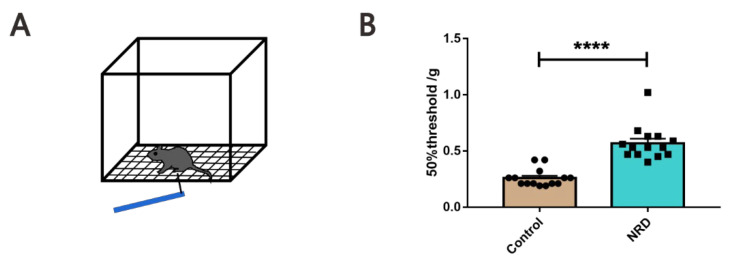
Declined pain sensitivity in NRD mice. (**A**) Schematic of von Frey filaments test. (**B**) Quantitative allodynia assessment by fifty percent paw withdrawal threshold testing. **** *p* < 0.0001 as determined by unpaired Student’s *t*-test. Data are expressed as the means ± SEMs. *n* (control) = 14, *n* (NRD) = 14.

The protocol for VFFT was referred to Chaplan SR, et al. to assess allodynia [22]. Mice were allowed to acclimate to plexiglass enclosures on top of a wire testing rack for 30 min before each test session (Figure 10A). After habituation, responses (withdrawal, shaking, or licking the paw) to mechanical stimulation in the middle area of the left hind paw were determined by applying the von Frey filaments using an up-down technique. The 50% threshold is calculated based on previous research [22].

#### 2.3.10. Food-Seeking Test

To evaluate the effect of zinc sulfate irrigation on the olfaction of mice, the food-seeking test at 1 and 7 days after nasal irrigation was performed as our previous described [17]. Briefly, 1 × 1-cm food particle was buried 3 cm underneath the bedding in the middle of the cage. After fasting for 24 h, subjects went into the cage to search for hidden food for 300 s. The latency to find the food and begin to eat was recorded. If the subject did not find the buried food within 300 s, the test was stopped and recorded for 300 s.

#### 2.3.11. Statistical Analysis

The data were analyzed using GraphPad Prism version 7.0. The values are presented as the mean ± SEM. A *p*-value < 0.05 was considered statistically significant. The statistical significance of the differences in the conditional fear test and social novelty test was assessed by two-way ANOVA followed by Bonferroni posttests. For the rest of the data, comparisons of the means between two groups were performed using the unpaired Student’s *t*-test. The data for each test are summarized in Table 1.

## 3. Results

### 3.1. Open Field Test

Exploratory behavior in a novel environment and general locomotor activity were assessed in an open field for 10 min (Figure 2A,B). The total distance covered within 10 min was (3171 ± 224) cm in the NRD group and (3499 ± 149.5) cm in the control group (Figure 2C). The average speed of the NRD group was (412.5 ± 46.51) cm/min, and that of the control group was (465 ± 28.35) cm/min (Figure 2D). The NRD mice did not achieve statistical significance for either distance traveled or moving speed in the open field (*p* = 0.2336, *p* = 0.3437, respectively; Figure 2C,D). The data pertaining to the distance traveled in the center zone or the number of entries can be used to analyze the anxiety behavior of mice. The total distance of activity within 10 min in the central zone in the NRD group was (399.6 ± 41.42) cm, and that of the control group was (514.9 ± 33.11) cm (*p* = 0.039, Figure 3E). The number of the NRD group central zone entries was (33 ± 3.063) times compared to (42.86 ± 1.83) times for the control group (*p* = 0.0104, Figure 2F).

### 3.2. Elevated O Maze

Next, the elevated O-maze test was performed to evaluate anxiety in mouse models (Figure 3A). Compared to the control mice, the NRD mice traveled a shorter distance (*p* = 0.0381, Figure 3B), spent less time (*p* = 0.0414, Figure 3C), and had fewer number of entries (*p* = 0.0438, Figure 3D) in the open arms.

### 3.3. Forced Swim Test

To further assess the depressive state of our mouse model, we utilized a forced swim test (Figure 4A). Interestingly, we found that the NRD mice spent less time struggling than the control mice (*p* = 0.0012, Figure 4B).

### 3.4. Y-Maze Test

To evaluate whether the sensory deprivation of nasal breathing might affect spatial working memory, we used the Y-maze spontaneous alternation test for further behavioral assessment (Figure 5A). No difference between NRD mice and control mice was detected for the total number of arm entries, indicating that the exploratory disposition of NRD mice was not altered compared to control mice (*p* = 0.6946, Figure 5B). However, the NRD mice showed a deficit in spontaneous alternation (*p* = 0.0276, Figure 5C).

### 3.5. Novel Object Recognition Test

We then assessed the declarative memory of mouse models using the object recognition test, which measures an animal’s ability to distinguish between novel and familiar objects (Figure 6A). After a retention phase of 1 h, the testing phase revealed that the NRD mice displayed a significantly lower discrimination ORT index than control animals (*p* = 0.0074, Figure 6B).

### 3.6. Fear Conditioning Test

The contextual and the tone-cued fear conditioning tests were used to evaluate conditional learning and memory. In the contextual conditioning test, compared with the state before and during training, the performance of NRD mice and control mice was similar, the freezing time was significantly increased (*p* < 0.0001, *p* < 0.0001, respectively; Figure 7B), but there was no significant difference (*p* > 0.9999, *p* > 0.9999, respectively; Figure 7B) between the NRD and control mice. Interestingly, in the memory retrieval phase, the NRD mice had a longer freezing time in contrast to the control mice (*p* = 0.0199, Figure 7C; *p* = 0.0436, Figure 7D). In the tone-cued fear conditioning test, NRD mice did not display deficits in freezing acquisition (*p* > 0.9999, Figure 8B), but they showed significant memory retrieval impairment following 1 h and 24 h after training (*p* = 0.0285, Figure 8C; *p* = 0.0233, Figure 8D).

### 3.7. Social Novelty Test

The social behavior was assessed using the three-chamber paradigm test (Figure 9A). The experiment consisted of three parts: training phase, social phase, and social novelty phase. During the social phase, both NRD and control mice showed similar performances, preferring to sniff stranger 1 versus sniffing the empty cage (*p* < 0.0001, *p* = 0.0008, respectively; Figure 9B), but NRD mice spent significantly less time in the interaction zone (*p* = 0.0054, Figure 9C). Consistently, during the subsequent social novelty test, both mice spent more time sniffing stranger 2 than stranger 1 (*p* = 0.0004, *p* = 0.0381, respectively; Figure 8D). However, the preference index (ratio of time sniffing stranger 2 vs. stranger 1) revealed that the NRD mice had a significantly decreased preference for stranger 2 (*p* = 0.0386, Figure 9E).

### 3.8. Von Frey Filaments Test

The von Frey filaments test was used to assess allodynia (Figure 10A). The 50% mechanical thresholds of the NRD group and the control group were 0.5686 ± 0.04063 g and 0.2593 ± 0.02058 g, respectively (*p* < 0.0001, Figure 10B). The data showed that the 50% mechanical threshold of the NRD group was significantly higher than that of the control group.

## 4. Discussion

Nasal breathing is a ubiquitous organizer of dynamics across multiple brain areas and is simultaneously involved in various behaviors and states [10,25]. In this present study, general locomotor activity, emotion, learning and memory, social interaction, and mechanical pain were evaluated in a ZnSO_4_-induced mouse model to explore the behavioral effects of NRD.

Breathing is affected by emotions, such as sadness, happiness, or anxiety [26]; in turn, breathing control can also improve emotions [27]. But whether the sensory deprivation of nasal breathing can impact emotion is not known. To that end, we assessed the depression and anxiety state of mouse models using the open field, elevated O maze, and forced swim tests [14,15,16].

In the open field test, we found that the distance and shuttle times of the NRD mice in the central area were reduced, but the total distance and average speed in the open field had no significant difference. Meanwhile, the cumulative time, distance, and number of shuttles of NRD mice in the elevated O-maze open arms decreased, indicating that NRD may cause anxiety-like behaviors rather than reduced motor activity. Similarly, the struggle time of forced swim was also reduced, indicating that NRD can also induce depression-like behavior. There are numerous lines of evidence demonstrating that PFC circuitry is dysregulated in depression and anxiety. These include alterations of structure, markers of glutamatergic and GABAergic neurotransmission, and connectivity with downstream structures [28]. Considering that previous studies have shown that respiratory rhythm could contribute to information processing in the frontal neuronal network [8,10], we speculate that the depression-anxiety-like behavior of the NRD mice might be caused by the dysregulation of the PFC neural network.

Nasal breathing has been linked to memory processes [12,29]. The hippocampus and amygdala are important brain areas associated with memory and are regulated by breathing signals [30]. In our study, NRD mice showed cognitive impairments in several learning and memory tests aimed to assess both hippocampal-dependent memory (Y maze, object recognition task, and the contextual fear conditioning) [17,18,19] and amygdala-dependent memory (the tone-cued fear conditioning test) [30]. Results showed that the correct rate of autonomous alternation in Y maze decreased in the NRD mice, which did not cause by exploratory disposition. The ORT index of the NRD mice in the object recognition task decreased significantly, which is consistent with the features in the human memory experiment when only mouth breathing was retained [29]. Interestingly, in the training phase of the contextual fear conditioning test, when the mice were given 0.3-mA foot shock, the freezing time of the NRD group showed no significant difference compared to the control group, indicating that the NRD mice could successfully acquire contextual fear. Moreover, an increase in freezing time was observed in a 1-h and 24-h contextual memory retrieval test. These results suggest that the sensory deprivation of nasal respiratory can enhance hippocampal-dependent fear memory retrieval without affecting fear memory formation. Taken together, the hippocampal-dependent memory deficiency observed indicates that NRD mice acquired cognitive anomalies.

More notably, during the acquisition phase of the cued fear conditioning test, 0.5-mA foot shock caused parallel fear in the NRD group and control group. However, the NRD mice showed less freezing time during cued memory retrieval at 1 h and 24 h after the training. Cued fear is amygdala-dependent memory, suggesting that the sensory deprivation of nasal respiratory did not affect amygdala-dependent fear memory formation but is only responsible for the cued memory retrieval, consistent with a previous report [31]. Studies have described that amygdala, not mPFC, plays a role in fear acquisition [32,33], but both play a role in fear expression [34,35]. Our research further supports the existence of two separate brain mechanisms controlling initiation and maintenance of freezing behavior independently.

As mentioned above, NRD can cause depression in mice, and depression can cause social withdrawal [36]. To investigate how NRD mice might behave, we performed a three-compartment test and found that the NRD mice showed both impaired social behavior and social novelty behavior. This could be due to a general change in cognition or a lack of interest in social novelty [37] or impairment of smell [38]. At the same time, we tested the mice using the von Frey filaments test and found that the pain sensitivity of olfactory impaired mice was decreased, indicating that nasal respiration plays a role in pain perception. Many brain areas are closely related to nasal breathing, such as the amygdala, somatosensory cortex, and prefrontal cortex [7,8,9], and are involved in the perception of pain [39,40,41]. Therefore, the decreased pain sensitivity caused by NRD may arise from abnormal neuron activity in these brain areas.

In summary, this mouse model of NRD showed a variety of behavioral phenotypic changes, including depression-like behavior, anxiety-like behavior, cognitive impairments, impaired social behavior, social novelty behavior, and pain perception, indicating that nasal breathing may alter related behaviors by affecting the neural activity of the brain. Hence, relevant symptoms caused by abnormal nasal breathing sensory should be concerned when nasal breathing is limited or OB undergoes pathological changes, including malfunctions of the olfactory epithelium due to infections, such as COVID-19 [42], as well as neurodegenerative diseases [43], such as Alzheimer’s disease, Parkinson’s disease, etc.

## 5. Conclusions

### Limitations

ZnSO_4_ nasal irrigation can impair the sense of smell of mice and will recover to a certain extent with time (Figure S1). For rodents that rely heavily on smell, this will lead to changes in some behavioral domains [44]. However, the odorant and the mechanical responses are mediated by a common cAMP cascade of olfactory sensory neurons [5]. Part of the reasons of behavioral changes caused by olfactory destruction, perhaps, is the destruction of nasal breathing perception. Therefore, the relationship between the two is also something that cannot be ignored in future research. Due to the regeneration characteristics of the olfactory epithelium, can the injured behavioral phenotype of the NRD mice be rescued following the recovery of the olfactory epithelium? Additionally, the difference of behavior may be partly related to the difference in endocrine status [45]. What is the difference in the behavioral changes of female mice between male mice after the sensory deprivation of nasal breathing? These have important guiding significance for related clinical symptoms and further researches are needed.

## Figures and Tables

**Table 1:** Summary of data from the behavioral experiments.

		Control		NRD		*p*-Value	df
		Mean ± SEM	*n*	Mean ± SEM	*n*		
OFT	Distance in open field	3499 ± 149.5	14	3171 ± 224	14	0.2336	26
	Moving speed in open field	465 ± 28.35	14	412.5 ± 46.51	14	0.3437	26
	Distance in center	514.9 ± 33.11	14	399.6 ± 41.42	14	0.0390	26
	Number of center squares entered	42.86 ± 1.83	14	33 ± 3.063	14	0.0104	26
EOM	Distance in open arms	443.6 ± 55.05	13	295.7 ± 38.91	13	0.0381	24
	Time in open arms	170.6 ± 17.67	13	121.8 ± 14.18	13	0.0414	24
	Number of open arms entered	91.38 ± 7.238	13	73.23 ± 4.512	13	0.0438	24
FST	Struggle times	69.96 ± 3.945	17	51.28 ± 3.468	17	0.0012	32
YMT	Number of arms	38.6 ± 4.498	15	41.14 ± 4.555	14	0.6946	27
	Spontaneous alternation	66.88 ± 3.128	15	54.88 ± 4.157	14	0.0276	27
NORT	ORT index (%)	24.56 ± 10.08	13	−18.98 ± 10.95	13	0.0074	24
FCT (freezing %)	Free	7.347 ± 3.237	20	12.22 ± 4.46	20	0.3826	38
	Train	33.96 ± 3.05	20	37.38 ± 3.791	20	0.4864	38
	After 1 h	30.5 ± 3.567	20	43.55 ± 4.01	20	0.0199	38
	After 24 h	28.52 ± 2.431	20	37.78 ± 3.809	18	0.0436	36
TCF (freezing %)	Free	4.883 ± 2.757	16	4.258 ± 1.788	16	0.8504	30
	Train	32.93 ± 4.959	16	34.26 ± 5.783	16	0.8619	30
	After 1 h	61.14 ± 2.89	15	48.71 ± 4.538	15	0.0285	28
	After 24 h	69.89 ± 4.398	15	54.38 ± 4.723	16	0.0233	29
SNT	Sociability; empty	49.52 ± 4.164	13	66.78 ± 7.364	13	0.0525	24
	Sociability; stanger1	130.6 ± 7.801	13	111.3 ± 8.946	13	0.1157	24
	Sociability; assessment	2.64 ± 0.2201	13	1.762 ± 0.1841	13	0.0054	24
	Social novelty; stranger1	71.12 ± 10.32	13	62.69 ± 6.437	13	0.4951	24
	Social novelty; stranger2	103.7 ± 8.037	13	93.97 ± 10.85	13	0.4763	24
	Social novelty; assessment	2.046 ± 0.1855	13	1.469 ± 0.1854	12	0.0386	23
VFFT	50% mechanical threshold	0.2593 ± 0.02058	14	0.5686 ± 0.04063	14	<0.0001	26

## Data Availability

The data provided in this study can be obtained from the corresponding author under reasonable request.

## Supplementary Materials

The following are available online at https://www.mdpi.com/article/10.3390/brainsci11121626/s1, Figure S1: Result shows that zinc sulfate irrigation will affect the sense of smell of mice, and will recover to a certain extent with time. *** *p* < 0.001, **** *p* < 0.0001, Control vs NRD; ### *p* < 0.001 NRD day 1 vs NRD day 7; Two-way ANOVA. *n* (control) = 12, *n* (NRD) = 12.
