# Supplementary material for: Effect of Sensory Deprivation of Nasal Respiratory on Behavior of C57BL/6J Mice
Source: Brain Sci. 2021 Dec 9;11(12):1626. doi: 10.3390/brainsci11121626 (PMC8699203; doi:10.3390/brainsci11121626)
Supplement: Supplementary file 1 [file brainsci-11-01626-s001.zip › brainsci-1471909-supplementary.pdf]

Supporting information for

Effect of Sensory Deprivation of Nasal Respiratory on Behavior of C57BL/6J Mice

Yongji Zhu 1,†, Yujing Ye 1,†, Chenyang Zhou 1,†, Siqi Sun 1, Jingjing Zhang 1, Zixuan Zhao 1, Tingting Sun 2,
Jing Li 3, Jing Yang 1, Weiyun Li 1,* and Shanshan Li 1,*

1 Department of Basic Medicine, School of Medicine, Zhejiang University City College, Hangzhou 310015, China; zyj2901142505@163.com (Y.Z.); yyj000426@163.com (Y.Y.); zhoucy9086@163.com (C.Z.); ssq44747@163.com (S.S.); hata6r@163.com (J.Z.); zzx001110@163.com (Z.Z.); yangjing@zucc.edu.cn (J.Y.)

2 College of Food Science and Pharmaceutical Engineering, Zaozhuang University, Zaozhuang 277160, China; suntingting1218@126.com

3 Institute of Neuroscience and Anatomy, School of Medicine, Zhejiang University, Hangzhou 310058, China; lijing851@zju.edu.cn

* Correspondence: liweiyun@zucc.edu.cn (W.L.); liss0220@zucc.edu.cn (S.L.); Tel.: +(86)-571-8801-5208 (S.L.)

† These authors contributed equally to this work.

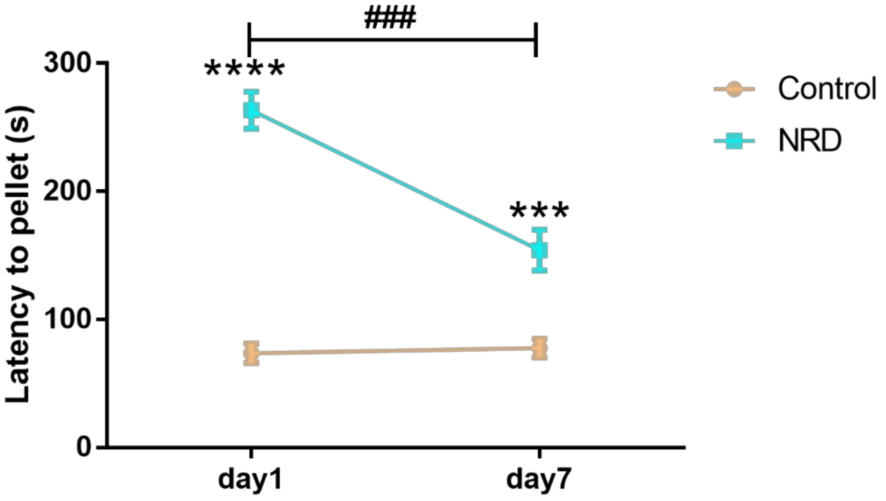

Figure S1.

To evaluate the effect of zinc sulfate irrigation on the olfaction of mice, the food-seeking test at 1 and 7 days after nasal irrigation was performed as our previous described [17]. Result shows that zinc sulfate irrigation will affect the sense of smell of mice, and will recover to a certain extent with time. ***$P < 0.001$, ****$P < 0.0001$, Control vs NRD; $^{###}P < 0.001$ NRD day1 vs NRD day7; Two-way ANOVA. n(control)=12, n(NRD)=12.
